# Immunomodulation and effects on microbiota after *in ovo* administration of chicken cathelicidin-2

**DOI:** 10.1371/journal.pone.0198188

**Published:** 2018-06-05

**Authors:** Tryntsje Cuperus, Marina D. Kraaij, Aldert L. Zomer, Albert van Dijk, Henk P. Haagsman

**Affiliations:** 1 Division of Molecular Host Defence, Department of Infectious Diseases & Immunology, Faculty of Veterinary Medicine, Utrecht University, Utrecht, The Netherlands; 2 Division Clinical Infectiology, Department of Infectious Diseases & Immunology, Faculty of Veterinary Medicine, Utrecht University, Utrecht, The Netherlands; Cincinnati Children’s Hospital Medical Center, UNITED STATES

## Abstract

Host Defense Peptides (HDPs) such as cathelicidins are multifunctional effectors of the innate immune system with both antimicrobial and pleiotropic immunomodulatory functions. Chicken cathelicidin-2 (CATH-2) has multiple immunomodulatory effects *in vitro* and the D-amino acid analog of this peptide has been shown to partially protect young chicks from a bacterial infection. However, the mechanisms responsible for CATH-2 mediated *in vivo* protection have not been investigated so far. In this study, D-CATH-2 was administered *in ovo* and the immune status and microbiota of the chicks were investigated at 7 days posthatch to elucidate the *in vivo* mechanisms of the peptide. In three consecutive studies, no effects on numbers and functions of immune cells were found and only small changes were seen in gene expression of Peripheral Blood Mononuclear Cells (PBMCs). In two studies, intestinal microbiota composition was determined which was highly variable, suggesting that it was strongly influenced by environmental factors. In both studies, *in ovo* D-CATH-2 treatment caused significant reduction of Ruminococcaceae and *Butyricicoccus* in the cecum and *Escherichia/Shigella* in both ileum and cecum. In conclusion, this study shows that, in the absence of an infectious stimulus, *in ovo* administration of a CATH-2 analog alters the microbiota composition but does not affect the chicks’ immune system posthatch.

## Introduction

Host Defense Peptides (HDPs) such as cathelicidins and defensins are important effector molecules of the innate immune system. These peptides are multifunctional as they show both broad-spectrum antimicrobial activity but also have many immunomodulatory functions [1,2]. In the search for alternatives to antibiotics, HDPs are considered promising candidates for both human and veterinary applications [3,4]. Initially the antimicrobial activity of HDPs was seen as their most important asset in their development as anti-infective drugs. However, antimicrobial activity of many HDPs is severely inhibited by components present in physiological environments such as salt or proteins [5–7]. Therefore, immunomodulatory activities are now believed to be essential for *in vivo* protection by these peptides and HDP analogs without antibacterial activity have been shown to provide protection against infections in animal models [8,9]. Chicken cathelicidin-2 (CATH-2) is one of the four cathelicidins of the chicken. Previously, a D-amino acid analog of this peptide (D-CATH-2) was described to protect young chickens against an *Escherichia coli* infection after administration into embryonated eggs (*in ovo*), a technique used commercially to administer vaccines to broiler eggs [10]. Immunomodulation is believed to be the mechanism behind this protection as direct antimicrobial activity is improbable due to the small amount of D-CATH-2 administered and the time gap of 10 days between peptide administration and onset of infection. Immunomodulatory activities of CATH-2 have been shown *in vitro*, including induction of chemokine production, inhibition of LPS-induced inflammatory mediators and enhancement of DNA-induced macrophage activation [11,12]. Similar to CATH-2, most immunomodulatory activities described for HDPs are identified by *in vitro* research. Therefore, the question remains which immunomodulatory mechanisms drive the anti-infective *in vivo* effects of CATH-2 and HDPs in general. In this study, we analyzed the immunomodulatory effect of *in ovo* administered D-CATH-2 by investigating the immune status of 7 day old chicks. This timepoint was chosen to correspond with our previous experiment in which we challenged chicks with *E*. *coli* at 7 days of age after *in ovo* administration with D-CATH-2 [10]. We investigated the number of immune cells in peripheral blood and organs and assessed the functionality of PBMCs and dendritic cells (DC). In addition, the effects of *in ovo* D-CATH-2 on the intestinal morphology and microbiota were determined.

## Materials and methods

### Synthesis of cathelicidin peptides

D-CATH-2 (all D-amino acid sequence: RFGRFLRKIRRFRPKVTITIQGSARF-NH_2_) was generated by solid-phase synthesis using Fmoc-chemistry and purified to >95% by RP-HPLC by CPC Scientific Inc. (Sunnyvale, USA).

### Animals

This study was conducted in accordance with the Dutch Experiments on Animals Act and conformed the European standards (European Directive 2010/63/EU). All separate animal experiments were approved by the Central Authority for Scientific Procedures on Animals (CCD) (Permit number: 2014.II.05.37). Ross 308 broiler eggs at day 18 of embryonic development (ed18) were obtained from a commercial hatchery (Lagerwey, Lunteren, The Netherlands). Chickens were housed in negative pressure HEPA isolators where temperature was gradually decreased from 35 °C to 27.5 °C over 7 days posthatch and fed a commercial broiler diet ad libitum (Research Diet Services, Wijk bij Duurstede, The Netherlands, no antibiotics or coccidostats added).

### *In vivo* experiments

Peptide preparation and injection was similar to Cuperus *et al*. [10]. Briefly, D-CATH-2 was dissolved in PBS (1.48 mM NaH_2_PO_4_.H_2_0, 8.06 mM Na_2_HPO_4_, 20 mM NaCl, pH 7.27, filter sterilized) to a concentration of 0.22 mg/ml, to which cholesterol (5% v/v, 2 mg/ml in absolute ethanol) was added. Three days before hatch (ed18, estimated embryo weight = 22 g), 1 mg/kg D-CATH-2 or buffer was administered *in ovo* into the amniotic cavity using a 1 inch, 21G needle injected up to the hilt. Posthatch, only male chickens were selected (n = 6-8/group) and housed in separate isolators per experimental treatment. Animals were euthanized by electrocution followed by cardiac puncture and organs, blood and intestinal content were collected. Three separate *in vivo* experiments were conducted in which the same batch of chicken feed was used.

### PBMC isolation and tissue single cell isolation

Peripheral blood mononuclear cells (PBMCs) were isolated from whole blood using a Ficoll gradient. Spleen and cecal tonsil single cell suspensions were made by gently pressing the tissue through a 70 μM cell strainer followed by a Ficoll gradient for spleen samples. Cells were frozen at -80 °C until further analysis.

### Whole blood and tissue flow cytometry

Whole blood samples were incubated with the following labelled antibodies: CD45-APC (clone LT40, dilution 1:200), CD3-PE (clone CT-3, dilution 1:200), KUL01-FITC (dilution 1:50, Southern Biotech, Birmingham, AL, USA) and unlabeled antibodies: Bu-1 (clone AV20, dilution 1:500), MHC-II (clone 2G11, dilution 1:500) (Southern Biotech), CD41/CD61 (clone 11C3, dilution 1:100) and CD40 (clone AV79, dilution 1:100) (AbD Serotec, Kidlington, UK) for 30 min at 4 °C. After washing, samples with unlabeled antibodies were subsequently incubated for 30 min with secondary BV421 or PerCP-Cy5.5-labelled rat-anti-mouse antibodies (clone RMG1-1, dilution 1:500/1:800 respectively, BioLegend, San Diego, CA, USA). Samples were washed and analyzed by flow cytometry (FACSCantoII, BD Biosciences, San Jose, CA, USA) using FlowJo analysis software. Absolute cell numbers were determined using BD Trucount absolute counting tubes (BD Biosciences) according to the manufacturer’s protocol. Cell suspensions from cecal tonsils and spleen were also stained with above-mentioned antibodies and protocol.

### Phagocytosis assay

PBMCs were defrosted and seeded in duplicate in black 96-well plates (5x10^6^ cells/well) in RPMI-1640 medium without Phenol Red. After 1 hour of incubation at 41 °C, medium was replaced with pHrodo Green *E*. *coli* bioparticles (ThermoFisher, Waltham, MA, USA) suspended in RPMI-1640 without Phenol Red. Fluorescence (488/520 nm) was measured for up to 6 hours in a microplate reader at 41°C.

### Oxidative burst assay

PBMCs were defrosted and seeded in black 96-well plates (1.5x10^6^ cells/well) in RPMI-1640 without Phenol Red containing 10% FCS (Bodinco, Alkmaar, The Netherlands) and 1% penicillin/streptomycin (Life Technologies, Paisley, UK). After overnight incubation at 41 °C, medium was replaced with RPMI-1640 containing the profluorescent probe 2’,7’-dichlorofluorescein-diacetate (DCFH-DA, 10 μg/ml, Sigma-Aldrich, Zwijndrecht, The Netherlands). Fluorescence (488/520 nm) was measured in a microplate reader at 41 °C for up to 3 hours.

### Mixed lymphocyte reaction

In an allogeneic mixed lymphocyte reaction (MLR), responder PBMCs (from healthy adult chickens) were labelled with 5 μM CFSE (carboxyfluorescein succinimidyl ester, BioLegend, San Diego, CA, USA) in PBS for 10 min at 37 °C. To quench the staining, RPMI-1640 medium containing 10% FCS was added. In a 96-wells plate, 1x10^5^ labelled responder cells were added to the PBMCs from the experimental chickens in ratios ranging from 8:1–1:64. In other experiments, 1x10^5^ responder PBMCs were added to bone marrow-derived dendritic cells from experimental chickens in ratios ranging from 2:1–1:64. Cell mixtures were incubated for 5 days at 41°C and analyzed by flow cytometry using FlowJo analysis software. The percentage of proliferation was calculated relative to the proliferation in responder cells alone.

### PBMC microarray

RNA (n = 6/group) was isolated from PBMCs using Trizol (Ambion, Paisley, UK) precipitation and purified using RNeasy columns (Qiagen, Venlo, The Netherlands). An automated system (Caliper Life Sciences, Hopkinton, MA, USA) was used to perform cDNA synthesis, labeling, quantification, quality control by Bioanalyzer and fragmentation, starting with 3 μg total RNA from each samples as previously described [13]. Per treatment, three samples were labeled with Cy5 and co-hybridized with reference RNA (pooled RNA from all experimental samples) labeled with Cy3. The remaining samples were labeled and hybridized the opposite way (Cy3-labeled RNA from experimental samples with Cy5-labeled reference RNA). Microarrays used were chicken whole genome gene expression arrays V2 (Agilent, Santa Clara, CA, USA) representing 42034 *Gallus gallus* 60-mer probes in a 4x44K layout. Probe sequences were re-annotated by BLAST searching against the chicken genome version 76.4 at ENSEMBL. Microarray hydridization and washing was performed with a HS4800PRO system with QuadChambers (Tecan, Männedorf, Switzerland) using 1000 ng, 1–2% Cy5/Cy3 labeled cRNA per channel. Slides were scanned on an Agilent G2565BA scanner. Data was extracted using Imagene 8.0 (BioDiscovery, Hawthorne, CA, USA) and Loess normalization was performed on mean spot intensities [14]. Gene-specific dye bias was corrected by a within-set estimate [15]. Differences in gene expression between treatment groups were analyzed by Linear Model for Microarray Analysis (LIMMA) with Benjamini-Hochberg FDR correction. Genes with p<0.05 and fold-change>2 were considered significantly changed. Because the Bioanalyzer results showed monospecific peaks in the cRNA pointing to contamination of the RNA, principal component analysis (PCA) was performed on log2 transformed expression values of genes with a >2-fold difference between samples. The principal component (PC2) with the highest correlation to the peaks from the Bioanalyzer results was then used as a covariate in LIMMA representing severity of contamination. Results of the corrected and uncorrected LIMMA analyses were compared to obtain significantly changed genes.

### Dendritic cell culture

Bone marrow was harvested from the chicken’s femurs and tibias and homogenized through a 70 μM cell strainer. Cells were centrifuged and resuspended in RPMI-1640 medium containing 5% chicken serum, 1% GlutaMAX-I and 0.5% penicillin/streptomycin (Life Technologies). Bone marrow cells were seeded at a concentration of 2.5x10^6^ cells/ml in 6-well plates and optimally diluted concentrations of recombinant chicken IL-4 and GM-CSF were added [16]. Plasmids were kindly provided by Prof. P. Kaiser and L. Rothwell (The Roslin Institute, Edinburgh, UK). Cells were cultured for 7 days at 41 °C, at days 3 and 5, medium was replaced with fresh medium containing IL-4 and GM-CSF. After harvesting at day 7, cells were stained with MHC-II, CD40 and CD86 (AbD Serotec, Kidlington, UK) antibodies and secondary BV421-labelled anti-mouse antibody as described above. Cells were washed and analyzed by flow cytometry and FlowJo analysis software.

### Villus/crypt ratio and goblet cell measurements

Tissue samples of duodenum, jejunum and ileum were fixed in 4% formaldehyde and paraffin-embedded. Tissue sections of 2 μm were stained with the combined Alcian Blue (pH 2.5)/PAS procedure according to Luna *et al* [17]. Microscopic images of representative cross-sections were analyzed using cellSens Dimension (Olympus, Tokyo, Japan) software. Per intestinal segment 5 representative and completely paired villus-crypt units were measured. The villus/crypt ratio was determined as the length of the villi divided by the depth of the mucosal crypt region. Size and density of Alcian Blue/PAS positive goblet cells was determined in 5 representative well oriented villi per intestinal sample.

### Microbiota analysis

Bacterial DNA was extracted from embryonic (ed18) intestinal samples (intestinal tissue including contents, harvested under sterile circumstances) and jejunal and cecal contents of 7 days old chicks using a phenol/bead-beating and magnetic bead separation method as described previously [18]. Per sample, 1 ng of DNA was amplified using F515/R806 primers (Caporaso et al, 2011) based on the V4 hypervariable region of the 16S rDNA gene (details of amplicon PCR described in Kozich et al, 2013). After amplification, samples were sequenced by Illumina MiSeq (Illumina Inc., San Diego, CA, USA) and processed using modules implemented in Mothur V1.31.2 [19]. Reads were trimmed using Btrim (Kong, 2011) with a sliding window size of 5 nt and an average quality score of 25 and contigs between read pairs were assembled. The sequences were aligned to the reference alignment SILVA SEED (release 102, www.mothur.org/wiki/Silva_reference_files). The ends of the sequences were trimmed in order that the sequences all started and ended at the same alignment coordinates. The resulting sequences were screened for chimeras using UCHIME (Edgar et al, 2011). Sequence data was subsampled to the lowest number of reads and sequences observed less than 10 times were removed. High quality aligned sequences were taxonomically classified using the Ribosomal Database Project-II naïve Bayesian classifier [20], requiring a 60% pseudobootstrap confidence score. Aligned sequences were subsequently clustered into operational taxonomic units (OTUs, defined by 97% similarity) using the average linkage clustering method.

PCA was performed on log transformed OTU abundances of all OTUs accounting for at least 1% of the reads in one sample. The relative abundance of OTUs and higher taxonomic levels were compared between treatment groups and statistically tested using the Mann-Whitney *U* test. The Benjamini-Hochberg FDR procedure was used for multiple testing correction.

### Statistics

Statistical analysis was performed using SPSS 22 software (IBM, Armonk, USA). In separate *in vivo* experiments, differences between treatment groups were tested using unpaired *t*-tests and Mann-Whitney *U* tests for non-normally distributed data. In addition, overall differences in variables measured in all three experiments (weight, whole blood leukocyte populations, phagocytosis, oxidative burst, VC ratio, goblet cell parameters) were analyzed using a General Linear Model. Differences were considered statistically significant if p<0.05.

## Results

### Chicken bodyweight

To investigate a possible effect of *in ovo* administration of a CATH-2 analog on chicken growth, animals were weighed at multiple timepoints posthatch. Bodyweight was not different between non-treated and D-CATH-2 treated animals (S1 Fig) at any timepoint.

### Leukocyte numbers in peripheral blood, spleen and cecal tonsils

**Fig 1:**
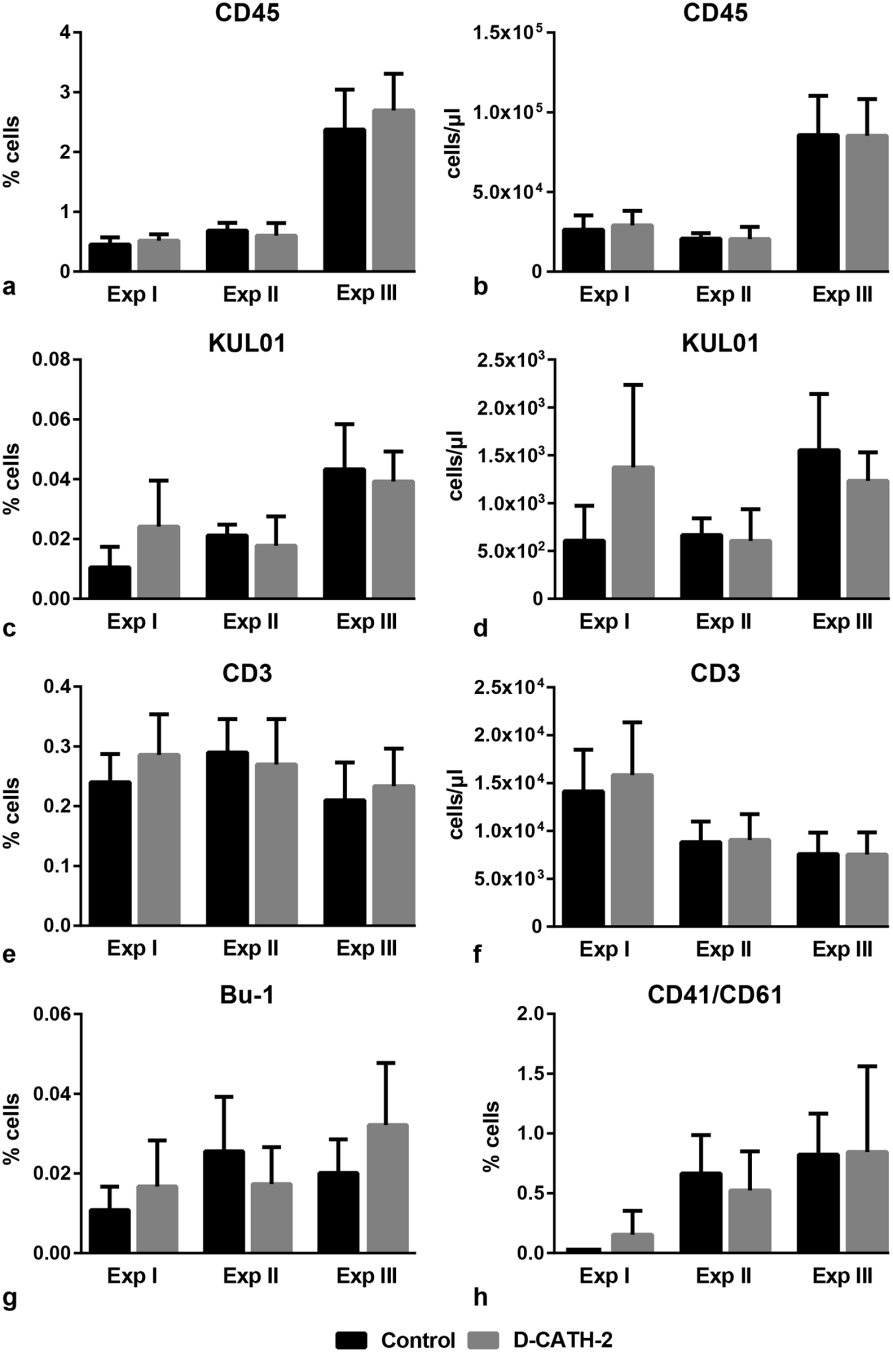
Leukocyte populations in peripheral blood. Data from three repeated experiments. (a)-(b) total leukocytes (CD45), (c)-(d) mononuclear phagocytes (KUL01), (e)-(f) T-cells (CD3), (g) B-cells (Bu-1), (h) thrombocytes (CD41/CD6). Depicted are mean percentages of total cells (a,c,e,g,h) or mean absolute cell numbers (b,d,f) ± s.d., n = 6-8/group. Data of independent experiments were analyzed by an unpaired *t*-test, while combined data were analyzed using a General Linear Model.

Leukocyte numbers were measured in peripheral blood to investigate a possible effect of *in ovo* administration of D-CATH-2 on the systemic immune status of the chickens. In addition to total leukocytes (CD45), populations of mononuclear phagocytes (KUL01), T-cells (CD3), B-cells (Bu-1) and thrombocytes (CD41/CD61) were determined (Fig 1). In Exp I, a trend towards an increase of KUL01 positive cells was seen in D-CATH-2 treated animals, both in percentage in whole blood (Fig 1c, p = 0.071) and in absolute cell numbers (Fig 1d, p = 0.068). However, in the two following experiments, differences in KUL01 positive cells were not observed. In the other measured leukocyte populations, no differences were seen in any of the experiments between control and peptide treated animals (Fig 1a, 1b, 1e–1h). In addition, expression of MHC-II and CD40, two cell surface proteins involved in antigen presentation, was measured on the KUL01 positive population in samples derived from Exp II. Expression of these markers was not different between control and D-CATH-2 treated animals (S2 Fig).

In samples of Exp II, leukocyte populations were also determined in spleen and cecal tonsils. None of the determined populations showed significant differences between control and D-CATH-2 treated animals (S3 Fig).

### Functional capacity of PBMCs

**Fig 2:**
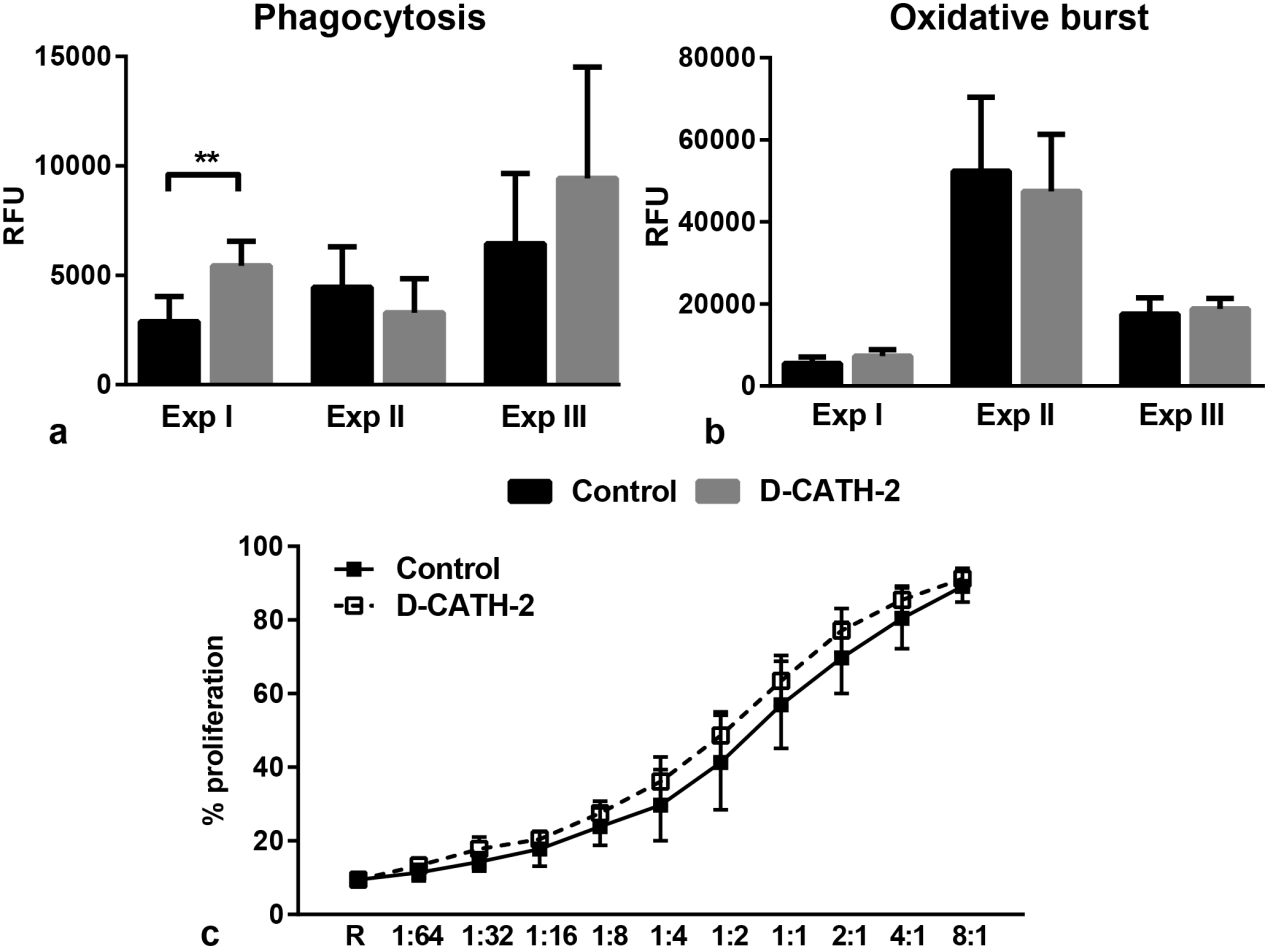
PBMC functions measured by *ex vivo* assays. (a) Phagocytosis measured by uptake of pHrodo Green labelled *E*. *coli*. Increase in fluorescence from 1–6 hours of incubation. Data from three repeated experiments, (b) Oxidative burst measured by conversion of DCFH-DA to fluorescent DCF. Increase in fluorescence in 3 hours of incubation. Data from three repeated experiments, (c) Proliferation of responder PBMCs measured by MLR, Samples from Exp I, R = responder cells alone. Depicted are mean ± s.d., n = 5-8/group, ** = p<0.01. Data from independent experiments were analyzed by an unpaired *t*-test, combined phagocytosis and oxidative burst data were analyzed by a General Linear Model.

To examine whether *in ovo* administration of D-CATH-2 could have an effect on PBMC function we analyzed phagocytosis and oxidative burst in samples from all three experiments and proliferative capacity in samples from Exp I. An increased capacity for phagocytosis was seen in PBMCs from D-CATH-2 treated animals in samples from Exp I (p = 0.002), while in the other two experiments no differences in phagocytosis were observed between treatment groups (Fig 2a). A similar response was found for oxidative burst; in PBMCs of Exp I a trend towards increased oxidative burst (p = 0.053) was seen in samples from D-CATH-2 treated chickens (Fig 2b). Samples from Exp II or III or overall results showed no difference. Stimulation of the oxidative burst by phorbol myristate acetate (PMA, 1 μg/ml) yielded similar results (S4 Fig). In addition, a mixed lymphocyte reaction (MLR) was performed to measure the capacity of PBMCs from the experimental chickens to activate T cells. The proliferation measured was similar in samples from D-CATH-2 treated chickens compared to control chickens (Fig 2c). These data indicate *in ovo* treatment with D-CATH-2 does not affect the functional capacity of PBMCs at 7 days posthatch.

### PBMC microarray

To analyze differences in gene expression in PBMCs from control and D-CATH-2 treated chickens, a whole genome microarray was performed on samples from Exp III. We observed 56 differentially expressed genes with a fold-change of more than 2. However, contamination of the RNA was observed, potentially caused by excessive amounts of globin mRNA (originating from red blood cells). PCA on gene expression differences revealed that 28 of the differentially expressed genes correlated with the observed RNA contamination with an R^2^>0.5, indicating that measured expression of these genes could be strongly influenced by the (globin) contamination. Interestingly, 85% (24/28) of these genes were downregulated in PBMCs from D-CATH-2 treated animals compared to the control treatment. The differentially expressed genes for which a gene description was available are shown in S1 Table.

### Phenotype and function of bone-marrow derived dendritic cells

**Fig 3:**
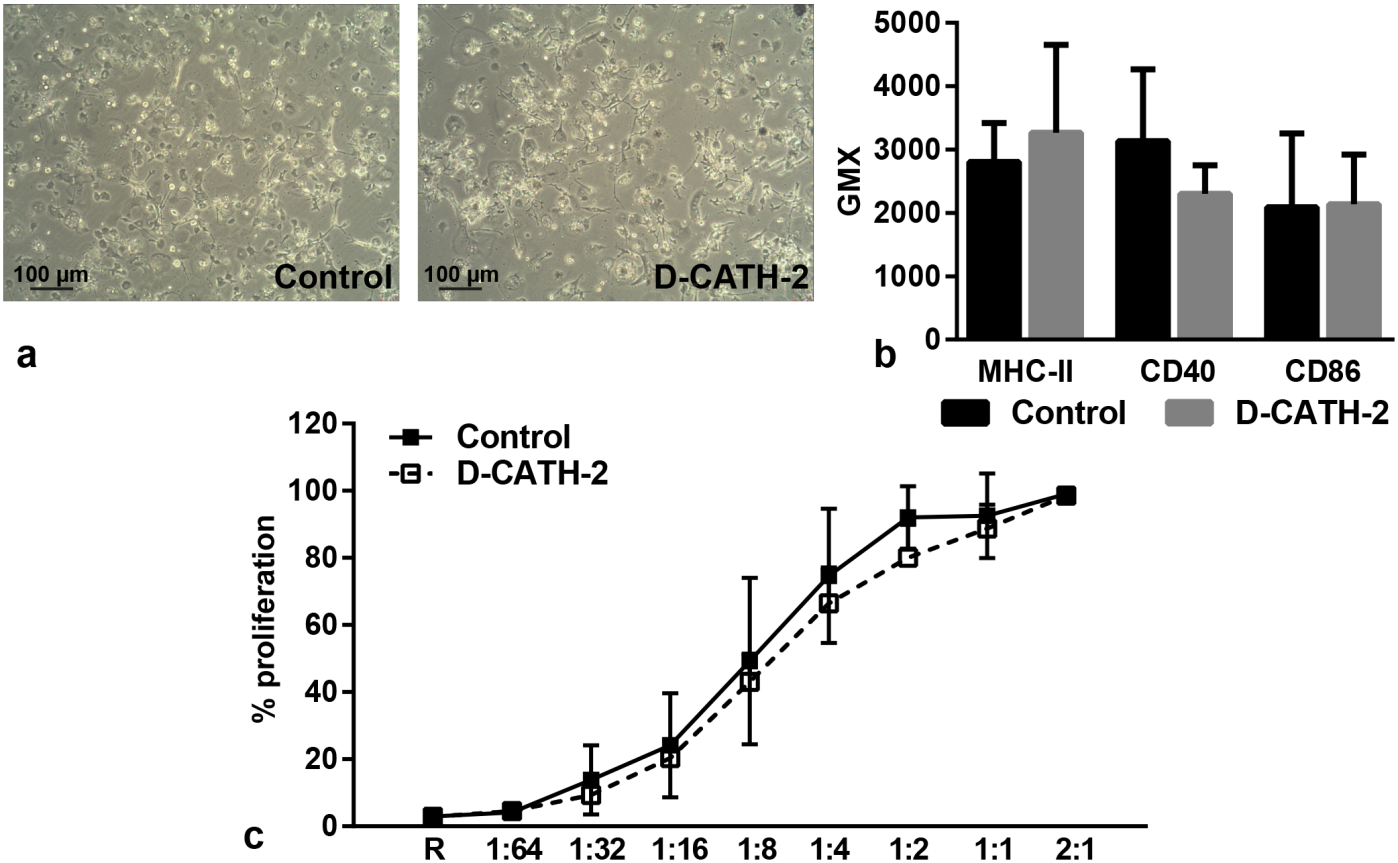
Phenotype and function of dendritic cells derived from Exp I. (a) Representative phenotype of bone-marrow derived DCs after 7 days of differentiation with IL-4 and GM-CSF (magnification: 100x), (b) MHC-II, CD40 and CD86 expression measured at day 7, (c) Proliferation of responder PBMCs measured by MLR, R = responder cells alone. Depicted are mean ± s.d., n = 5-6/group. Data were analyzed by an unpaired *t*-test.

In addition to PBMCs, bone-marrow derived dendritic cells from samples of Exp I were analyzed to determine the effect of *in ovo* administration of D-CATH-2 on this cell type. After 7 days of differentiation, DC phenotype was not different between cells derived from control chickens or chickens treated with D-CATH-2 (Fig 3a). In addition, expression of cell surface markers MHC-II, CD40 and CD86 was similar in cells from both treatment groups (Fig 3b). In an MLR of DCs with responder PBMCs, no difference was observed between the proliferative capacity of DCs from control chickens or from chickens treated with D-CATH-2 (Fig 3c).

### Intestinal architecture and goblet cell parameters

**Fig 4:**
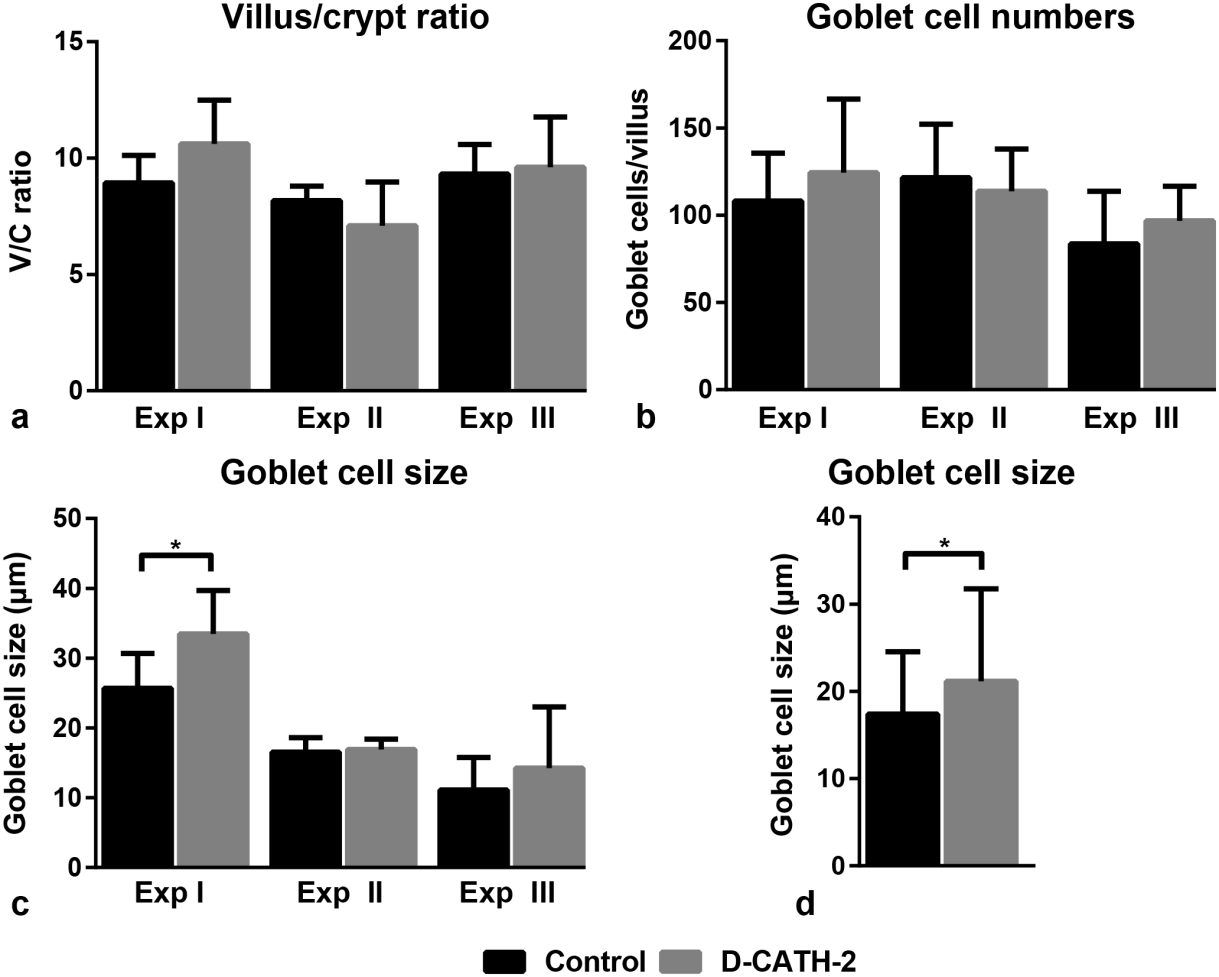
Intestinal architecture and goblet cell parameters in duodenum of 7 day old chicks. Data from three repeated experiments. (a) Villus/crypt ratio, (b) Goblet cell numbers per villus, (c) Goblet cell size, (d) Goblet cell size, combined data from three repeated experiments. Depicted are mean ± s.d., n = 6-7/group, * = p<0.05. Data were analyzed by an unpaired *t*-test, combined data were analyzed using a General Linear Model.

The ratio of villus height to crypt depth (VC ratio) was measured to determine the effect of *in ovo* administration of D-CATH-2 on intestinal development. In the duodenum, VC ratio was not different between intestinal samples from control or treated chickens in any of the three experiments (Fig 4a). In addition, numbers and size of goblet cells were determined as a measure of intestinal mucus production. Goblet cell numbers were not affected by D-CATH-2 in the duodenum in any of the experiments (Fig 4b). Goblet cell size in the duodenum was increased in D-CATH-2 treated animals in Exp I (p = 0.037, Fig 4c) and also significantly increased when the data from three experiments were combined (p = 0.036, Fig 4d). VC ratio or goblet cell parameters were not different between treatment groups in the ileum or jejunum (S5 Fig).

### Microbiota

**Fig 5:**
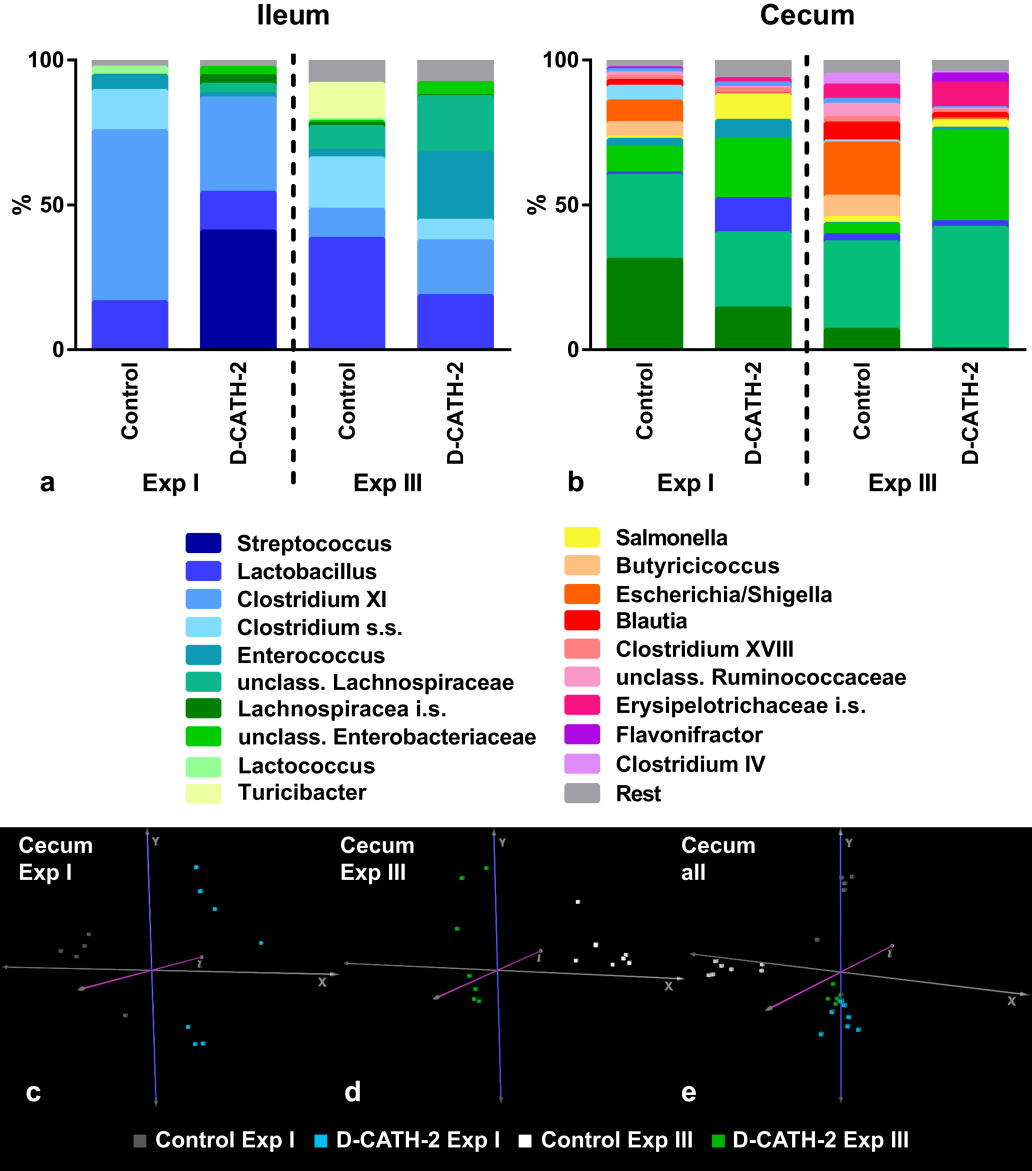
Microbiota at 7 days of age. Major bacterial genera in the chicken ileal (a) and cecal (b) content (n = 5-8/group). Depicted are the genera present at >1% average relative abundance across all treatment groups in their respective location. PCA plots of the bacterial communities in individual samples. (c) cecal microbiota in Exp I, (d) cecal microbiota in Exp II, (e) combined cecal microbiota data from Exp I and III.

Microbiota composition in the ileum and cecum was determined in samples from Exp I and III (Fig 5a and 5b). In addition, DNA from embryonic intestinal samples (ed18) was analyzed to determine the possible presence and composition of microbiota at the time of *in ovo* injection. However, bacterial DNA concentrations in the embryonic intestines were extremely low and far below the minimal input for sequencing, indicating no measurable microbiota was present in these samples. The global differences in bacterial composition of the treatment groups were analyzed by performing principal component analysis (PCA) on OTU abundances. In the analysis of the separate experiments, a clear separation could be seen between samples of the different treatment groups (cecum: Fig 5c and 5d, ileum: Panels a and b in S6 Fig). However, when data from the two experiments were combined, samples from control or D-CATH-2 treated groups did not cluster, instead all four analyzed groups clustered separately, most strongly in the cecum (Fig 5e, Panel c in S6 Fig). These results indicate that the composition of the microbiota is not only affected by treatment, but also by other influences such as environment considering that the treatment groups were housed separately.

**Fig 6:**
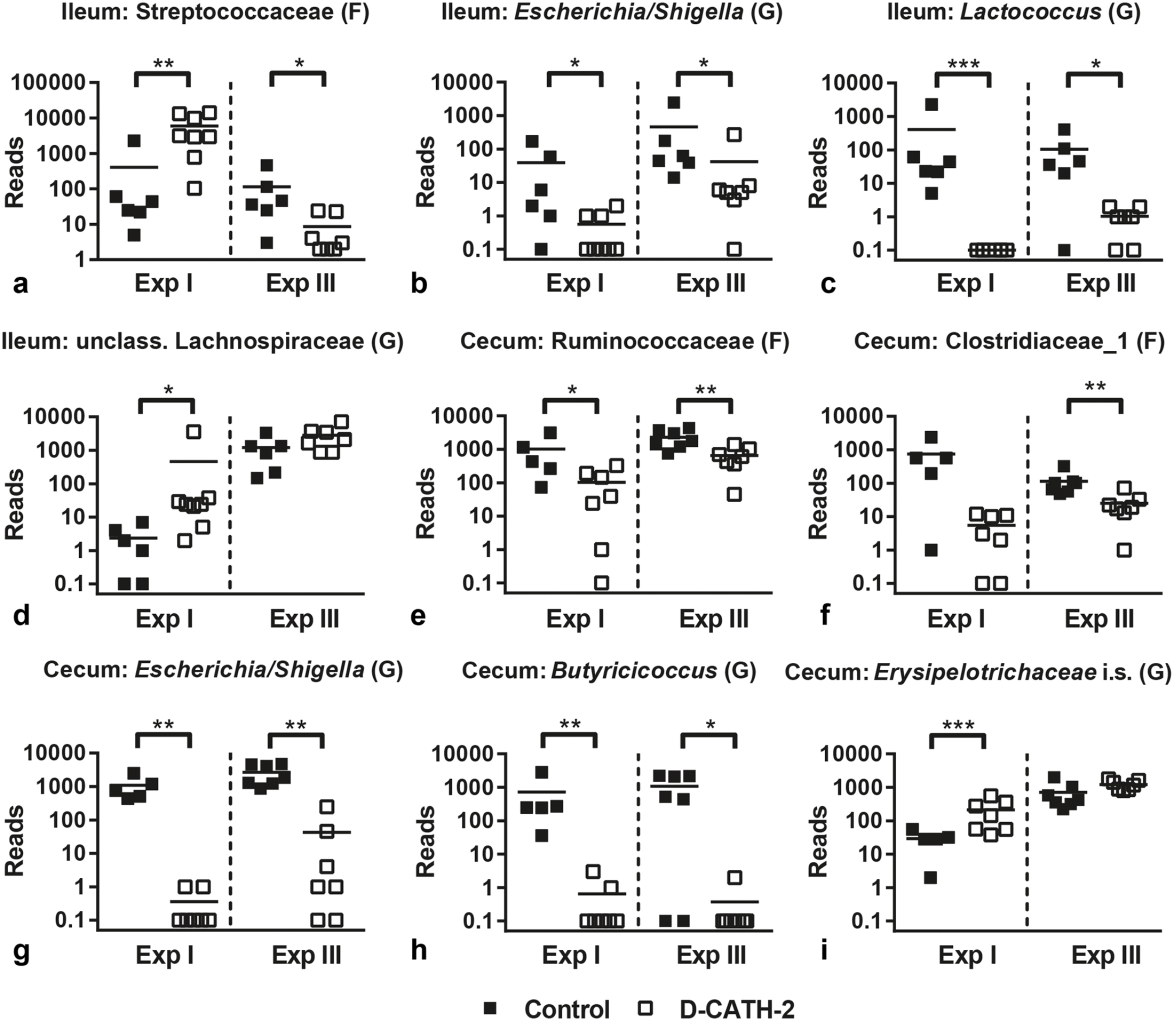
Relative bacterial abundance in selected families (F) and genera (G). Ileum: (a) Streptococcaceae, (b) *Escherichia/Shigella*, (c) *Lactococcus*, (d) unclassified Lachnospiraceae. Cecum: (e) Ruminococcaceae, (f). Clostridiaceae_1, (g) *Escherichia/Shigella*, (h) *Butyricicoccus*, (i) *Erysipelotrichaceae* incertae sedis. Reads were normalized to total number of reads per sample. * = p<0.05, ** = p<0.01, *** = p<0.001. Data were analyzed using the Mann-Whitney *U* test.

The influence of group environment was also apparent in observed differences in relative bacterial abundance. Between control and CATH-2 treatment groups, in both Exp I and III many significant differences were observed; however, these were experiment specific. Only a small number of bacterial families and genera were affected by D-CATH-2 treatment in both studies (Fig 6).

In the ileum, the abundance of the family of Streptococcaceae (Lactobacillales) was different between treatments in both experiments, but the results showed contrasting directions in Exp I (p = 0.004) and Exp III (p = 0.011) (Fig 6a). At the genus level, *Escherichia/Shigella* (Enterobacteriaceae) was reduced by D-CATH-2 treatment in both experiments (p = 0.029 and 0.022 respectively, Fig 6b). *Lactococcus* abundance was decreased by D-CATH-2 in both experiments (p = 0.0006 and 0.013 respectively, Fig 6c). The abundance of unclassified Lachnospiraceae was increased in the ileum in Exp I (p = 0.007) and showed a trend towards increase in Exp III (p = 0.063, Fig 6d).

In the cecum, two families of Clostridiales; Ruminococcaceae (p = 0.028 for Exp I and p = 0.006 for Exp II) and Clostridiaceae_1 (p = 0.060 for Exp I and p = 0.006 for Exp II) were affected by D-CATH-2 treatment in both experiments (Fig 6e and 6f), though reduction of Clostriaceae_1 in Exp I was not significant. At the Genus level, similar to ileum, *Escherichia/Shigella* (Enterobacteriaceae) was reduced (p = 0.002 and 0.002 respectively, Fig 6g). D-CATH-2 reduced *Butyricicoccus* (Ruminococcaceae) in both experiments (p = 0.002 and 0.020 respectively, Fig 6h). Finally, *Erysipelotrichaceae* incertae sedis in the cecum was increased in D-CATH-2 treated animals in Exp I (p = 0.009) and showed a trend towards increase (p = 0.064) in Exp III as well (Fig 6i).

Alpha diversity was not reproducibly different between treatment groups in either cecum or ileum (S7 Fig). In Exp I, differences in OTU numbers were not seen between control and D-CATH-2 treated chickens, while in Exp III OTU numbers were significantly lower in the cecum of D-CATH-2 treated animals compared to control animals (Panel a of S7 Fig). Similar differences between experiments were obtained with a Shannon diversity analysis (Panel b of S7 Fig).

## Discussion

The study described here aimed to elucidate the mechanisms behind the protection of young chicks against colibacillosis after *in ovo* administration of the HDP analog D-CATH-2 which we described in a previous article [10]. Multiple parameters reflecting immune status were measured and in addition, the influence of CATH-2 *in ovo* administration on intestinal morphology and microbiota was analyzed.

Evidence of *in vivo* immunomodulatory effects of HDPs was previously shown in mouse models. Firstly, IDR-1, an HDP analog with no *in vitro* antimicrobial activity was reported to protect mice from infections with multiple bacterial pathogens through effects on monocytes and macrophages [9]. HDP administration was also shown to protect against endotoxic shock by decreasing LPS-induced inflammatory mediators [21,22]. In our study, *in ovo* D-CATH-2 administration did not reproducibly affect the measured immune parameters, including leukocyte numbers in blood and organs and functionality of PBMCs and DCs. In addition, gene expression of PBMCs showed only very limited changes in our study. The main difference between the studies mentioned and our set of experiments is the presence or absence of an infectious stimulus. Whereas in our previous *in vivo* study with D-CATH-2 in infected animals changes in leukocyte numbers were apparent [10], the present study focused on the effect of CATH-2 administration on the immune system in unstimulated animals, which might explain the lack of observed effects. Similarly, uninfected mice treated intratracheally with LL-37 or IDR-1 showed no changes in lung cytokine responses [23] and uninfected CRAMP knockout mice have similar blood and lung leukocyte numbers as wildtype mice [24,25]. Moreover, in two studies in which bacterially derived cationic peptides were fed to chickens, effects on *ex vivo* leukocyte functions were only seen if an additional stimulus such as PMA or CpG was present [26,27]. However, in the present study we saw no change in PBMC oxidative burst even when stimulated by PMA. Finally, although all chickens used in our experiments appeared healthy, presence of an undetected infectious stimulus could have led to the effects on KUL01 cell numbers and phagocytosis in Exp I, while these effects were absent in Exp II and III. A possible mechanism explaining differences in CATH-2 effects between naïve and stimulated animals could be so-called trained immunity. This process was previously shown for several stimuli such as whole *C*. *albicans* or β-glucan and works through epigenetic reprogramming of cells [28,29]. To successfully decipher *in vivo* immunomodulatory mechanisms of CATH-2 in the future, studies comparing naïve and stimulated animals are needed and additionally epigenetic analyses should be performed. Independent of the mechanism, the absence of an immune response in naïve treated animals is positive for drug development as it indicates this peptide has no pro-inflammatory side effects.

The villus/crypt ratio of the intestine is a measure for intestinal functionality and development and measurements of goblet cell size and numbers may give information about intestinal mucus production. These parameters undergo drastic changes in the developing chick, both pre- and posthatch [30–32]. The parameters can be negatively affected by infections of intestinal pathogens such as *Salmonella*, while several feed additives like butyrate and mannanoligosaccharide are able to positively influence intestinal morphology [33–36]. Villus/crypt ratio and goblet cell numbers were not affected by *in ovo* administration of D-CATH-2, showing that *in ovo* administration of this CATH-2 analog has no deleterious effect on intestinal development. In addition, a small increase of goblet cell size in the duodenum in response to D-CATH-2 administration was seen, which could indicate D-CATH-2 might increase mucus production. LL-37 has previously shown to increase mucus production *in vitro* [37]. Similar to the immunomodulatory effects, *in ovo* administration of D-CATH-2 might show more effect on these parameters in the presence of an infectious stimulus. Further studies should be conducted to investigate whether CATH-2 analogs could protect against pathogen-induced intestinal damage as was recently shown for administration of a cathelicidin analog in piglets with post-weaning diarrhea [38].

The intestinal microbiota is strongly linked to host immunity, both influencing and being influenced by the immune system [39–41]. Since in our studies D-CATH-2 was administered during embryonic development, the effect of this peptide on microbiota development was investigated. Embryos have long been considered sterile, but this paradigm is challenged by putative reports of bacteria in amniotic fluid and fetal membranes [42]. In embryonated chicken eggs, bacteria were also detected by culture-based methods [43]. However, in the present study using a sequencing-based approach, no detectable microbiota was found in 18-day old chicken embryos.

Large differences in microbiota composition were observed between two separate experiments and clustering of samples also correlated to housing of the chickens, indicating that microbiota was more strongly influenced by environmental factors than by CATH-2 treatment. The so-called ‘cage effect’ is a well-known confounding factor in microbiota studies in mice [44], one study estimated cage to contribute for 31% to the variance in murine microbiota composition [45]. Like mice, chickens are coprophagic animals; thus, a strongly shared microbiota within one cage is to be expected. In addition, large differences in microbiota composition were previously described between three repeated chicken trials [46]. Commercial chickens hatched in hatcheries are not colonized by microbiota derived from their mothers like in mammals but pick up their initial flora from the environment or handlers, leading to highly variable microbiota development. These results emphasize the importance of repeated trials to show the real effect of an examined treatment on microbiota composition.

The HDPs α-defensin 5 and LL-37 were previously shown to have an effect on microbiota composition in rodents [47,48]. In our study, D-CATH-2 was able to reproducibly affect abundance of a number of bacterial families and genera in both the ileum and cecum. Diversity of the microbiota was not affected by D-CATH-2 in contrast to regular antibiotics which decrease overall diversity of chicken microbiota and also reduce *Lactobacillus* and promote Clostridia species [49]. The most clear-cut result of D-CATH-2 administration was the reduction of *Escherichia/Shigella* in both ileum and cecum. *Escherichia/Shigella* are potential pathogens and abundance of this genus was found to correlate negatively with growth and fat digestibility in broiler chickens [50]. High numbers of *E*. *coli* in mouse microbiota were also shown to correlate to higher susceptibility to colonization by *Salmonella* [51]. Finally, *Escherichia/Shigella* was implicated in the pathogenesis of inflammatory bowel disease in both mouse models and human studies [52–54]. Reduction of *Escherichia/Shigella* might therefore be considered a positive effect of D-CATH-2 administration. Interestingly, both chickens administered yeast overexpressing the porcine cathelicidin PMAP-36 and piglets with diarrhea treated with a snake cathelicidin showed reduced *E*. *coli* counts in respectively cecal content and feces [38,55], indicating that effects on this genus could be a more general HDP effect. In addition to the effect on *Escherichia/Shigella*, D-CATH-2 treatment also reproducibly reduced abundance of Ruminococcaceae and the genus *Butyricicoccus* belonging to this Family. *Butyricicoccus* and Ruminococcaceae in general are butyrate producing bacteria and considered beneficial members of the microbiota. Reduction of Ruminococcaceae abundance is correlated with diarrhea in both humans and dogs [56,57]. D-CATH-2 administration thus seems to have both beneficial and possibly unwanted influences on the intestinal microbiota. Additional work is needed to clarify these effects and the possible functional consequences on chicken performance and immune function.

In conclusion, *in ovo* administration of CATH-2 analogs showed little effect on the immune status of naïve chickens 7 days posthatch, suggesting that an infective stimulus is needed for the effect of the peptides to become apparent. However, D-CATH-2 treatment did affect several bacterial taxa, indicating that modulation of the gut microbiota might be one of the *in vivo* mechanisms of D-CATH-2.

## Supporting information

S1 Fig Chicken bodyweight over the course of the experiment. Weight curve from combined data from the three repeated experiments (n = 6–11 chickens/group in each experiment); depicted are mean ± s.d. Data of independent experiments were analyzed by an unpaired t-test, overall data were analyzed using a General Linear Model.
(TIF)

S2 Fig Expression of MHC-II and CD40 on KUL01 positive peripheral blood mononuclear phagocytes derived from Exp II. (a) MHC-II, (b) CD40. Depicted are mean fluorescence ± s.d., n = 6/group. Data were analyzed by an unpaired *t*-test.
(TIF)

S3 Fig Leukocyte populations in cecal tonsils and spleen from Exp II. (a) cecal tonsils, (b) spleen. Total leukocytes (CD45), mononuclear phagocytes (KUL01), T-cells (CD3), B-cells (Bu-1), thrombocytes (CD41/CD61). Depicted are mean percentages of cells ± s.d., n = 6-7/group, n.d. = non-detectable. Data were analyzed by an unpaired *t*-test.
(TIF)

S4 Fig Oxidative burst of PBMCs stimulated with PMA. Oxidative burst (stimulated with 1 μg/ml PMA) measured by conversion of DCFH-DA to fluorescent DCF. Increase in fluorescence in 3 hours of incubation. Data from three repeated experiments. Depicted are mean ± s.d., n = 5-8/group. Data from independent experiments were analyzed by an unpaired *t*-test, combined data were analyzed by a General Linear Model.
(TIF)

S5 Fig Intestinal architecture and goblet cell parameters in ileum and jejunum of 7 day old chicks. Data from three repeated experiments. (a)-(b) Villus/crypt ratio, (c)-(d) Goblet cell numbers per villus, (e)-(f) Goblet cell size. Depicted are mean ± s.d., n = 6-7/group. Data were analyzed by an unpaired *t*-test, combined data were analyzed using a General Linear Model.
(TIF)

S6 Fig PCA plots of the bacterial communities in individual ileal samples. (a) ileal microbiota in Exp I, (b) ileal microbiota in Exp III, (c) combined ileal microbiota data from Exp I and III.
(TIF)

S7 Fig Measures of alpha diversity of ileal and cecal microbiota. (a) Richness as defined by number of OTUs, (b) Shannon diversity index which takes into account both relative abundance and evenness of species in a sample. * = p<0.05, ** = p<0.01. Data were analyzed by unpaired *t*-test or Mann-Whitney *U* test in the case of non-normally distributed data.
(TIF)

S1 Table Results of microarray analysis. Differentially expressed genes between PBMCs from chickens treated with D-CATH-2 and control. Data were analyzed by Linear Model for Microarray Analysis (LIMMA) with Benjamini-Hochberg FDR correction.
(PDF)
