# Supplementary figures and images for: Immunomodulation and effects on microbiota after in ovo administration of chicken cathelicidin-2
Source: PLoS One. 2018 Jun 5;13(6):e0198188. doi: 10.1371/journal.pone.0198188 (PMC5988267; doi:10.1371/journal.pone.0198188)

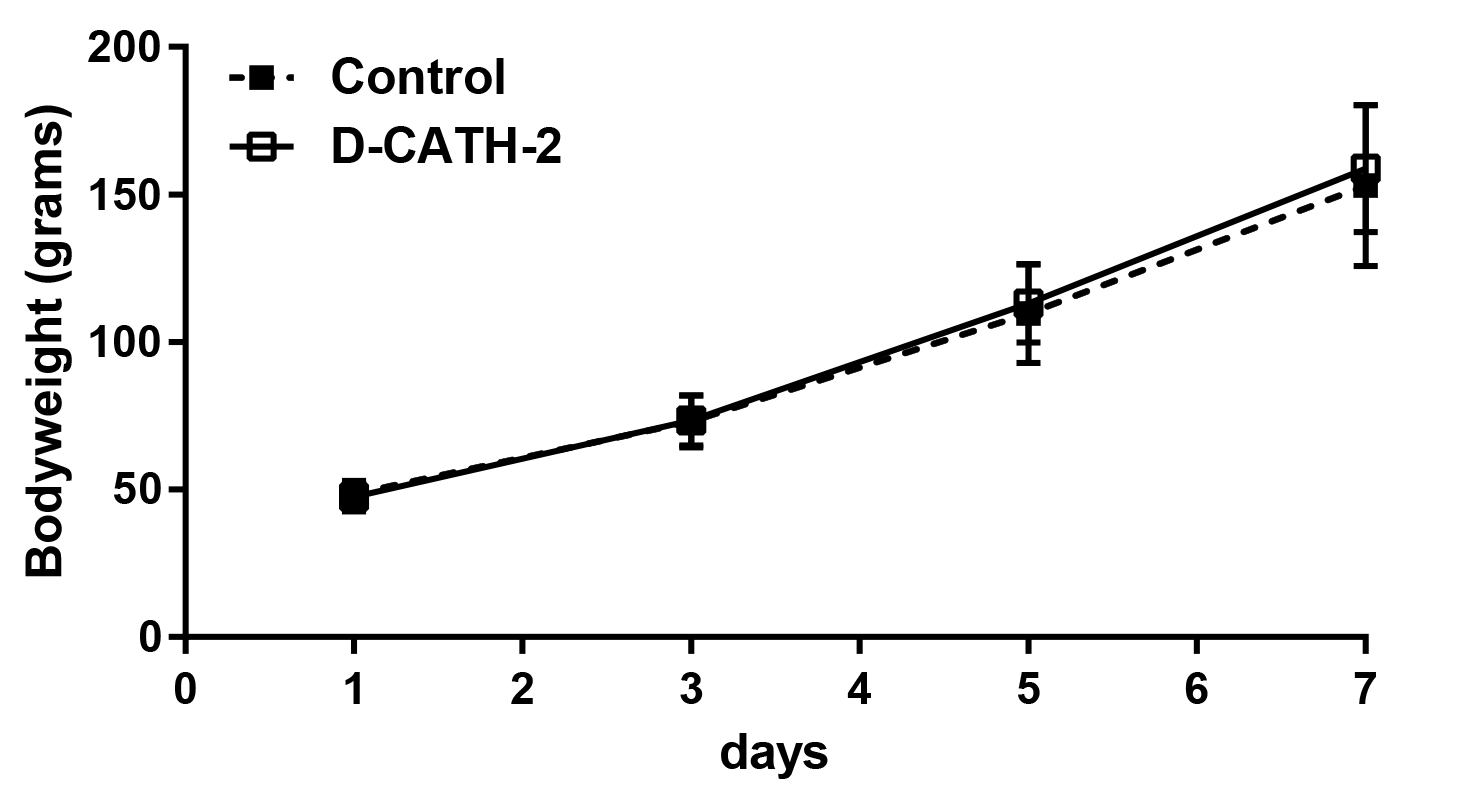

Supplement: S1 Fig — Weight curve from combined data from the three repeated experiments (n = 6–11 chickens/group in each experiment); depicted are mean ± s.d. Data of independent experiments were analyzed by an unpaired t-test, overall data were analyzed using a General Linear Model. (TIF) [file pone.0198188.s001.tif]

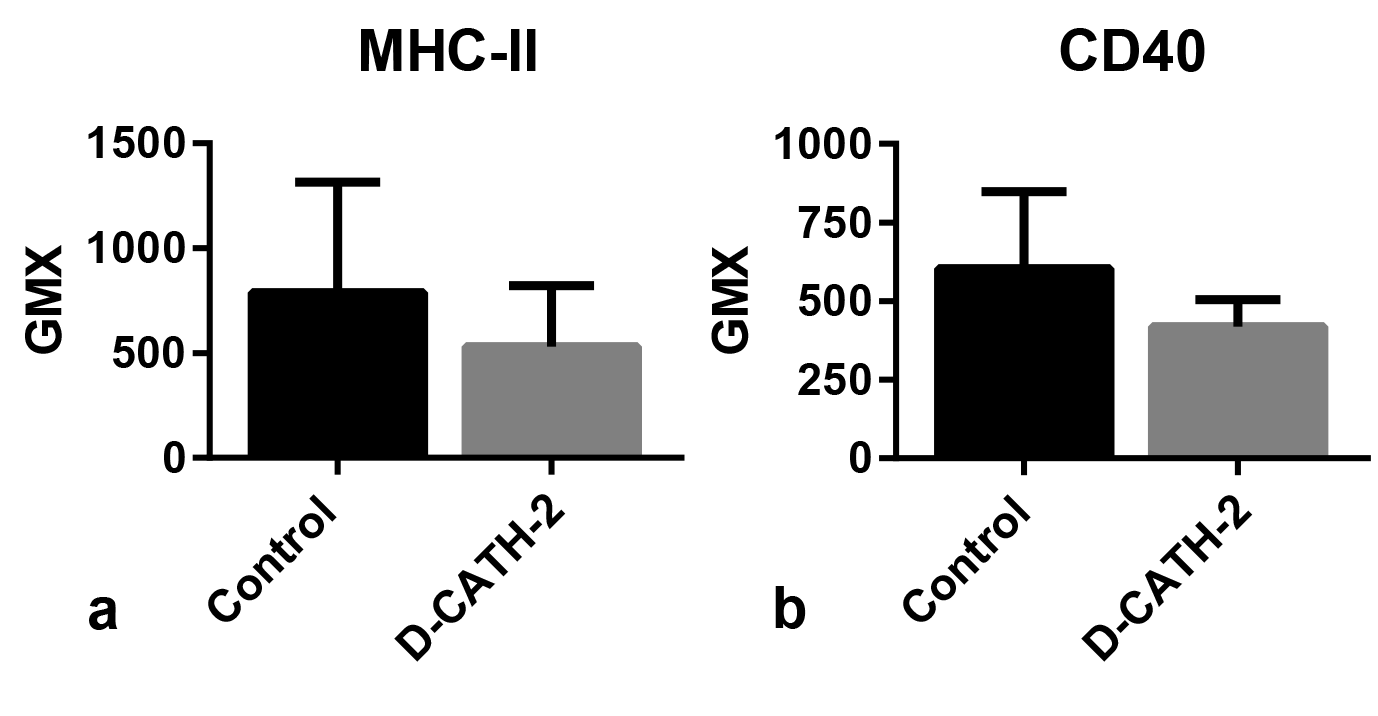

Supplement: S2 Fig — (a) MHC-II, (b) CD40. Depicted are mean fluorescence ± s.d., n = 6/group. Data were analyzed by an unpaired t-test. (TIF) [file pone.0198188.s002.tif]

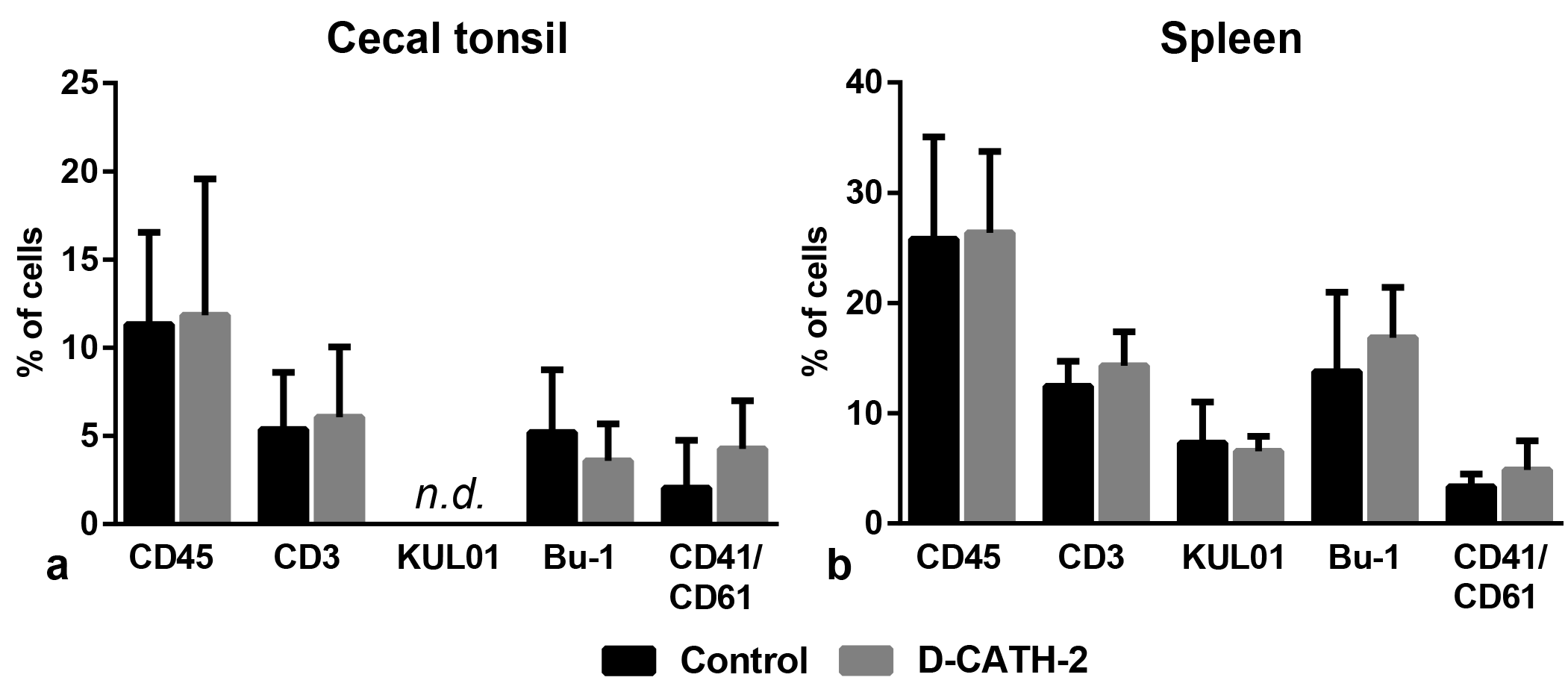

Supplement: S3 Fig — (a) cecal tonsils, (b) spleen. Total leukocytes (CD45), mononuclear phagocytes (KUL01), T-cells (CD3), B-cells (Bu-1), thrombocytes (CD41/CD61). Depicted are mean percentages of cells ± s.d., n = 6-7/group, n.d. = non-detectable. Data were analyzed by an unpaired t-test. (TIF) [file pone.0198188.s003.tif]

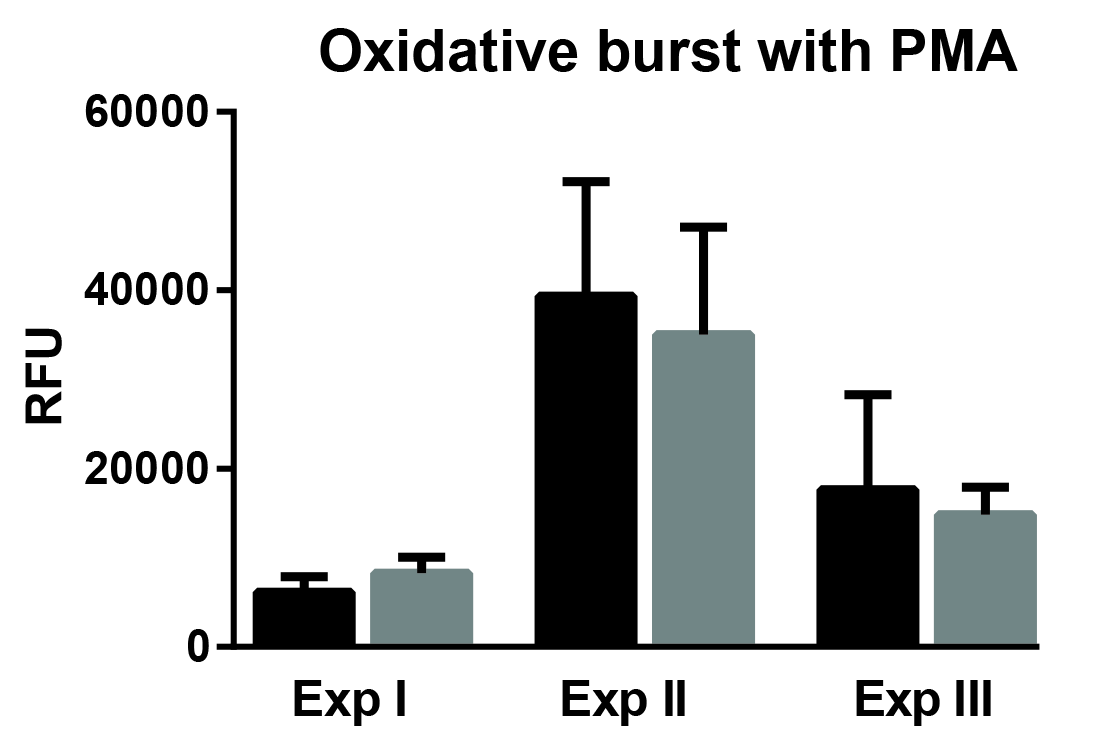

Supplement: S4 Fig — Oxidative burst (stimulated with 1 μg/ml PMA) measured by conversion of DCFH-DA to fluorescent DCF. Increase in fluorescence in 3 hours of incubation. Data from three repeated experiments. Depicted are mean ± s.d., n = 5-8/group. Data from independent experiments were analyzed by an unpaired t-test, combined data were analyzed by a General Linear Model. (TIF) [file pone.0198188.s004.tif]

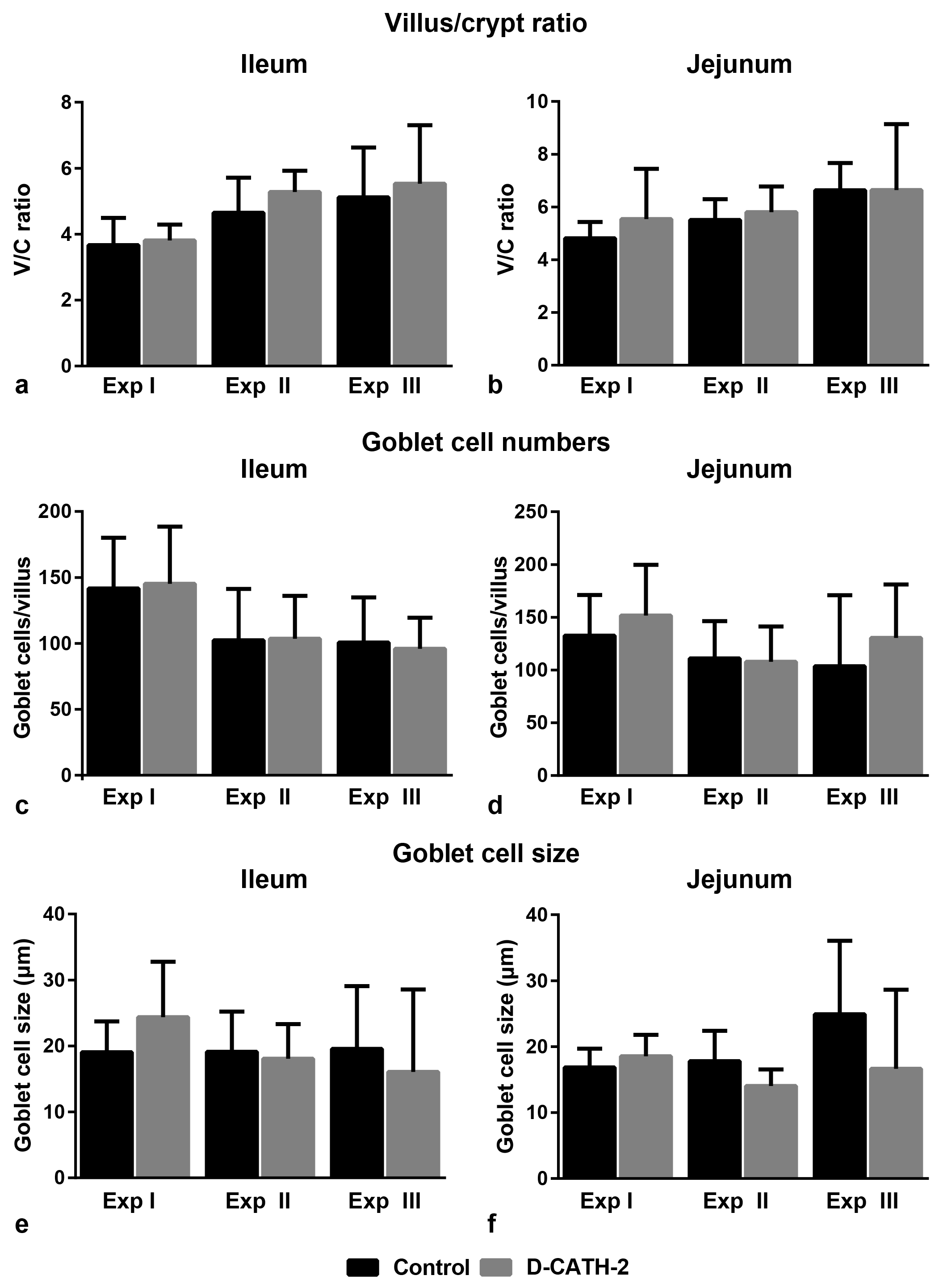

Supplement: S5 Fig — Data from three repeated experiments. (a)-(b) Villus/crypt ratio, (c)-(d) Goblet cell numbers per villus, (e)-(f) Goblet cell size. Depicted are mean ± s.d., n = 6-7/group. Data were analyzed by an unpaired t-test, combined data were analyzed using a General Linear Model. (TIF) [file pone.0198188.s005.tif]

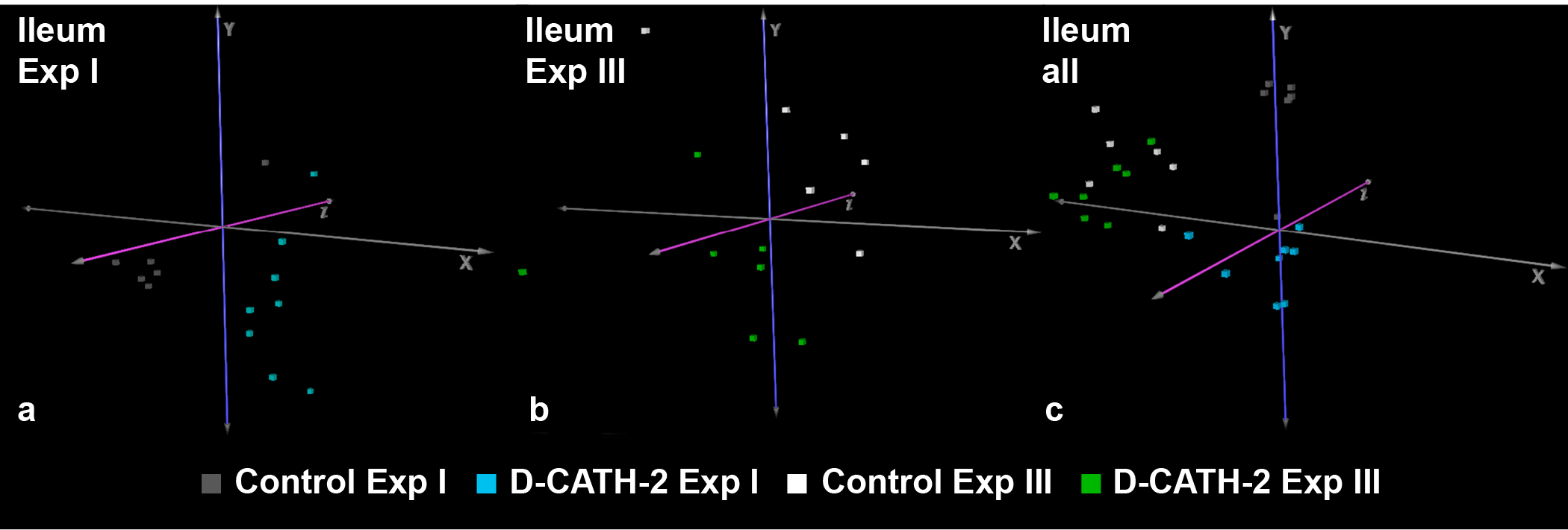

Supplement: S6 Fig — (a) ileal microbiota in Exp I, (b) ileal microbiota in Exp III, (c) combined ileal microbiota data from Exp I and III. (TIF) [file pone.0198188.s006.tif]

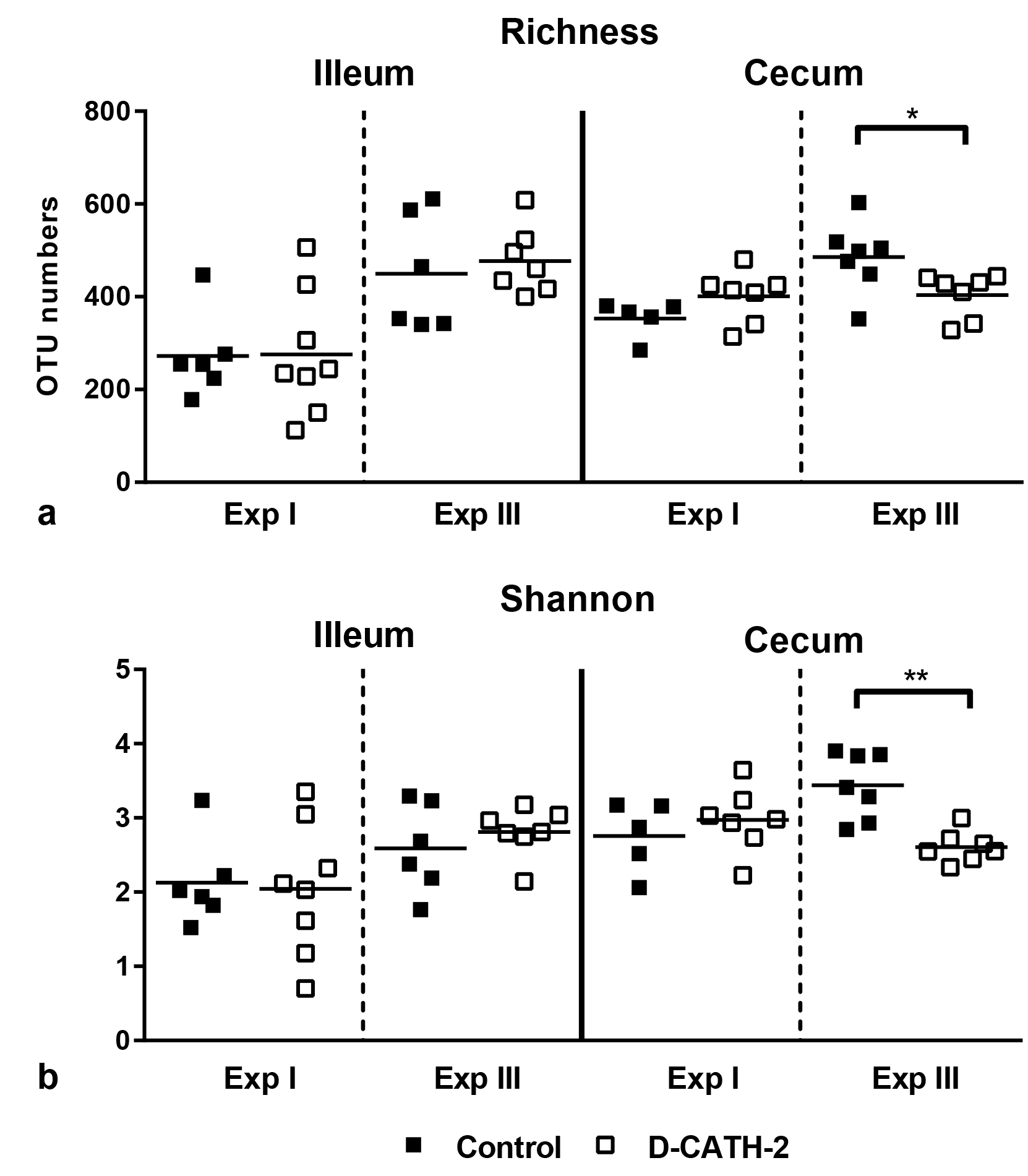

Supplement: S7 Fig — (a) Richness as defined by number of OTUs, (b) Shannon diversity index which takes into account both relative abundance and evenness of species in a sample. * = p<0.05, ** = p<0.01. Data were analyzed by unpaired t-test or Mann-Whitney U test in the case of non-normally distributed data. (TIF) [file pone.0198188.s007.tif]
