# Supplementary material for: Immunomodulation and effects on microbiota after in ovo administration of chicken cathelicidin-2
Source: PLoS One. 2018 Jun 5;13(6):e0198188. doi: 10.1371/journal.pone.0198188 (PMC5988267; doi:10.1371/journal.pone.0198188)
Supplement: S1 Table — Differentially expressed genes between PBMCs from chickens treated with D-CATH-2 and control. Data were analyzed by Linear Model for Microarray Analysis (LIMMA) with Benjamini-Hochberg FDR correction. (PDF) [file pone.0198188.s008.pdf]

Table S1 Results of microarray analysis.
Differentially expressed genes between PBMCs from chickens treated with D-CATH-2 and control. Data were analyzed by Linear Model for Microarray Analysis (LIMMA) with Benjamini-Hochberg FDR correction.

Gene description	***Fold change D-CATH-2 vs control***	***p-value***	***R^2 contamination***
COL4A3; type IV collagen alpha 3 chain	2.48	0.044	0.178
AMACR; alpha-methylacyl-CoA racemase	2.15	0.029	0.058
ISCA1; iron-sulfur cluster assembly 1 homolog	0.50	0.037	0.082
SRP72 ; signal recognition particle 72 kDa protein	0.48	0.008	0.356
ANKHD1; ankyrin repeat and KH domain-containing protein 1	0.37	0.039	0.001
C3ORF55; PQ Loop Repeat containing 2-like	0.35	0.001	0.292
ITGB4; integrin beta-4	0.35	0.001	0.408
KCNA1; potassium channel, voltage gated shaker related subfamily A	0.30	0.017	0.001
JARID2; jumonji, AT rich interactive domain 2	0.29	0.021	0.013
NUTF; nuclear transport factor 2	0.29	0.016	0.002
IGSF11; immunoglobulin superfamily, member 11	2.77	0.043	0.770
GRIA4; glutamate receptor, ionotropic, AMPA 4	2.47	0.020	0.846
LRP6; low density lipoprotein receptor-related protein 6	2.32	0.011	0.757
VRK2; vaccinia related kinase 2	2.28	0.032	0.786
MARK3; MAP/microtubule affinity-regulating kinase 3	2.26	0.011	0.785
PTPN13; protein tyrosine phosphatase, non-receptor type 13	2.23	0.004	0.600
CRMP1B; collapsin response mediator protein 1	2.16	0.038	0.584
CNNM4; cyclin and CBS domain divalent metal cation transport mediator 4	2.06	0.040	0.702
DST; dystonin	2.06	0.039	0.723
RAB44; Ras-related protein Rab-3D	2.05	0.038	0.716
ACOX2; acyl-coenzyme A oxidase	2.05	0.041	0.717
RILPL1; Rab interacting lysosomal protein-like 1	2.03	0.049	0.752
CPEB2; cytoplasmic polyadenylation element binding protein 2	2.02	0.018	0.728
MGLUR5B; metabotropic glutamate receptor 5	2.00	0.037	0.657

Uncharacterized genes (32) are not displayed in this table
